# Altered HDL proteome predicts incident CVD in chronic kidney disease patients

**DOI:** 10.1016/j.jlr.2021.100135

**Published:** 2021-10-09

**Authors:** Baohai Shao, Anna V. Mathew, Carissa Thornock, Subramaniam Pennathur

**Affiliations:** 1 Department of Medicine, UW Medicine Diabetes Institute, University of Washington, Seattle, WA, USA; 2 Division of Nephrology, Department of Internal Medicine, University of Michigan, Ann Arbor, MI, USA; 3 Molecular and Integrative Physiology, University of Michigan, Ann Arbor, MI, USA

**Keywords:** CVD, case-control study, CKD, HDL, HDL-C levels, HDL proteomics, MS, matched logistic regression analysis, parallel reaction monitoring, APOA1, apolipoprotein A-I, APOA4, apolipoprotein A-IV, APOC3, apolipoprotein C-III, CKD, chronic kidney disease, CPROBE, Clinical Phenotyping and Resource Biobank Core, CRP, C-reactive protein, eGFR, estimated glomerular filtration rate, ESRD, end-stage renal disease, HD, hemodialysis, OR, odds ratio, PON1, paraoxonase/arylesterase 1, PON3, paraoxonase/arylesterase 3, PRM, parallel reaction monitoring

## Abstract

Patients with chronic kidney disease (CKD) are at high risk for CVD. However, traditional lipid risk factors, including low HDL levels, cannot completely explain the increased risk. Altered HDL proteome is linked with both CVD and CKD, but the role of HDL proteins in incident CVD events in patients with CKD is unknown. In this prospective case-control study, we used targeted proteomics to quantify 31 HDL proteins in 92 subjects (46 incident new CVD and 46 one-to-one matched controls) at various stages of CKD. We tested associations of HDL proteins with incident CVD using matched logistic regression analysis. In the model fully adjusted for clinical confounders, lipid levels, C-reactive protein, and proteinuria, no significant associations were found for HDL-C, but we observed inverse associations between levels of HDL proteins paraoxonase/arylesterase 1 (PON1), paraoxonase/arylesterase 3 (PON3), and LCAT and incident CVD. Odds ratios (per 1 SD) were 0.38 (0.18–0.97, *P* = 0.042), 0.42 (0.20–0.92, *P* = 0.031), and 0.30 (0.11–0.83, *P* = 0.020) for PON1, PON3, and LCAT, respectively. Apolipoprotein A-IV remained associated with incident CVD in CKD patients in models adjusted for clinical confounders and lipid levels but lost significance with the addition of C-reactive protein and proteinuria to the model. In conclusion, levels of four HDL proteins, PON1, PON3, LCAT, and apolipoprotein A-IV, were found to be inversely associated with incident CVD events in CKD patients. Our observations indicate that HDLs' protein cargo, but not HDL-C levels, can serve as a marker—and perhaps mediator—for elevated CVD risk in CKD patients.

Chronic kidney disease (CKD) associates with an up-to-30-fold increased risk for CVD compared with the general population, and CVD is the primary cause of morbidity and mortality in patients with CKD (1). However, traditional lipid or cardiovascular risk factors do not explain the high incidence of CVD frequently seen in this population. For example, statin use has limited utility for reducing CVD events in CKD (2), suggesting that factors other than LDL-C are relevant to atherogenesis in this disease. One important factor could be altered levels of HDL-C or its constituent proteins, as low HDL-C is a common feature in patients with CKD (3).

HDL is a circulating and noncovalent assembly of amphipathic proteins and lipids. Clinical and epidemiological studies show a robust and inverse association of HDL-C levels with CVD risk in the general population (4). Moreover, apolipoprotein A-I (APOA1), the major protein of HDL, is strongly antiatherogenic in animal models of hypercholesterolemia (5, 6). However, several lines of evidence suggest that genetically low concentrations of HDL-C do not causally associate with a high risk of CVD (7, 8). Moreover, elevating HDL-C in randomized clinical trials has not reduced CVD risk (9), suggesting that this metric does not reflect the proposed cardioprotective effects of HDL.

Animal studies provide strong evidence that one key cardioprotective effect of HDL is the promotion of cholesterol efflux from artery wall macrophages (5, 6). HDL particle number is another important and independent atheroprotective property of HDL. In human studies, both cholesterol efflux capacity of serum HDL (10, 11, 12, 13) and HDL particle number (14, 15) strongly and inversely associate with prevalent atherosclerosis and incident CVD events, and the associations remain significant after adjustment for other cardiovascular risk factors, including HDL-C. However, it is unclear whether HDL proteome is associated with incident CVD.

HDL carries a wide array of proteins that influence its cardioprotective properties. The major HDL protein is APOA1, which accounts for ∼70% of its protein mass. MS-based proteomic analysis has demonstrated that about 80 proteins can be reproducibly detected in HDL (16). These proteins are linked to lipoprotein metabolism, inflammation, protease inhibition, and complement regulation (17), suggesting that this cargo contributes to the anti-inflammatory and antiatherogenic properties of HDL. Importantly, we previously showed that the HDL proteome is markedly remodeled in dialysis patients (18). We also demonstrated that an abnormally low concentration of the antiatherosclerotic protein paraoxonase/arylesterase 1 (PON1) in HDL associates with both albuminuria and atherosclerosis as measured by coronary artery calcification in patients with type 1 diabetes (19).

Although low HDL-C levels are associated with CKD status, (20) it is unclear whether HDL proteins associate with incident CVD events in CKD patients. This information would be extremely useful in understanding the mechanisms underlying the development of CVD in CKD and for developing a biomarker that predicts CVD in this vulnerable population. In the current study, we determined whether the HDL proteome is associated with future CVD events in patients at different stages of CKD. Four HDL proteins—PON1, paraoxonase/arylesterase 3 (PON3), LCAT, and apolipoprotein A-IV (APOA4)—are associated inversely with incident CVD events in subjects with CKD. Our observations indicate that the protein cargo of HDL can serve as a marker—and perhaps mediator—for increased CVD risk in CKD patients.

## Materials and methods

### Subjects and experimental design

Established under the auspices of the George O’Brien Kidney Center at the University of Michigan, the Clinical Phenotyping and Resource Biobank Core (CPROBE) is a multicenter cohort of 1,235 adult individuals with CKD (21) with high-quality biologic specimens and clinical data stored for future translational research. Inclusion criteria for this study included age older than 18 years and stages 1–5 CKD (according to the CKD Epidemiology Collaboration creatinine equation). We performed a prospective case-control study by analyzing stored plasma samples from 92 CPROBE subjects with no prior history of CVD with and without incident CVD events (N = 46 each group; Table 1). CVD outcome is defined as myocardial infarction, angina, coronary artery bypass grafting or angioplasty/stenting of a coronary artery, stroke, peripheral arterial disease, congestive heart failure or arrhythmia self-reported by patients and confirmed with electronic health record or by International Classification of Diseases-9 code review at CPROBE and non-CPROBE sites. The frequencies of CVD endpoint outcomes for the incident CVD group are shown in supplemental Table S1. We used plasma samples collected at the time of enrolment (between January 2009 and July 2015) and prospective clinical data available to us at the time of this study (follow-up of 1–10 years). The 46 cases were individually matched to a control subject of the same sex and diabetic status in a one-to-one ratio. Both the CPROBE ancillary studies committee and the Institutional Review Board at the University of Michigan approved the analysis of these samples in a deidentified and blinded manner for HDL isolation and proteomic analysis.

**Table 1:** Clinical characteristics of subjects

Covariate	Control	Incident CVD	*P*
Number of subjects	46	46	
Age (years)	55.3 (31–79)	54.2 (31–81)	0.68^a^
Gender (female)	31 (67.4)	31 (67.4)	1.00^b^
BMI (kg/m^2^)	31.9 (27.2–37.1)	33.3 (27.5–39.2)	0.44^c^
White	31 (67.4)	33 (71.7)	0.64^b^
SBP (mm Hg)	133.0 (121–140)	135.5 (118–147.8)	0.55^c^
DBP (mm Hg)	71.0 (65–77)	73.0 (65.5–84)	0.22^c^
Hypertension	33 (71.7)	35 (76.1)	0.64^b^
Ever a smoker	18 (39.1)	16 (34.8)	0.67^b^
Present smoker	5 (10.9)	7 (15.2)	0.54^b^
Diabetes	16 (34.8)	16 (34.8)	1.00^b^
Proteinuria	4 (8.7)	9 (19.6)	0.14^b^
Glomerular disease	16 (34.8)	15 (32.6)	1.00^b^
eGFR (ml/min/1.73 m^2^)	44.3 (33.6–70.2)	43.9 (26.9–79.0)	0.53^c^
Serum creatinine (mg/dl)	1.4 (1.1–1.7)	1.6 (1.0–2.3)	0.45^c^
CKD stage			
1 and 2 (eGFR ≥ 60)	15 (32.6)	16 (34.8)	0.83^b^
3 (30 ≤ eGFR < 60)	21 (45.7)	12 (26.1)	0.050^b^
4 and 5 (eGFR < 30)	10 (21.7)	18 (39.1)	0.070^b^
UPC ratio	0.91 (0.19–3.49)	2.86 (0.54–5.65)	<0.0001^c^
CRP (mg/dl)	0.315 (0.080–0.623)	0.450 (0.146–1.300)	0.058^c^
Statin use	26 (56.5)	18 (39.1)	0.095^b^
HDL-C (mg/dl)	46 (39–56.8)	45.5 (35.5–51.5)	0.24^a^
LDL-C (mg/dl)	98.9 (75.5–113.4)	108.3 (86.6–123.3)	0.14^c^
Total cholesterol (mg/dl)	181 (163.5–201.8)	196 (168.3–219.3)	0.14^c^
Triglycerides (mg/dl)	146.5 (120.5–212)	199 (123–238)	0.19^c^

BMI, body mass index; DBP, diastolic blood pressure; SBP, systolic blood pressure; UPC ratio, urinary protein to creatinine ratio.

Entries are median (interquartile range) for continuous covariates and N (%) for categorical covariates. We matched cases to controls for gender and diabetic status in a one-to-one ratio.

a *P* values are from a Student’s *t-*test for normally distributed variables.

b *P* values are from a Chi-square test for categorical variables.

c *P* values are from Mann-Whitney *U* test for abnormally distributed variables.

### Laboratory measurements

Hypertension was defined as systolic blood pressure >140 mm Hg, diastolic blood pressure >90 mm Hg, or the use of antihypertensive drugs. Standard enzymatic methods were used to measure lipid profiles. Estimated glomerular filtration rate (eGFR) was calculated from serum creatinine measured on an ADVIA 2400 analyzer using the Jaffe reaction and calculated using the CKD Epidemiology Collaboration creatinine equation for adults. Glomerular disease included diabetic kidney disease, focal segmental glomerulosclerosis, membranous glomerulopathy (membranous nephropathy), IgA nephropathy, vasculitis, immune-complex glomerulonephritis, and lupus nephritis.

### Isolation of HDL

HDL (density 1.063–1.210 g/ml) was isolated by sequential ultracentrifugation from rapidly thawed plasma (19), using buffers supplemented with 100 μM diethylenetriaminepentaacetic acid and a protease inhibitor cocktail (Sigma, St. Louis, MO). The protein concentration of HDL was determined using the Lowry assay (Bio-Rad), with albumin as the standard.

### Digestion of HDL

Following the addition of freshly prepared methionine (5 mM final concentration) in 20% acetonitrile and 100 mM NH_4_HCO_3_, 5 μg of HDL proteins were reduced with dithiothreitol and then alkylated with iodoacetamide. After adding 0.2 μg of isotope-labeled [^15^N]APOA1 (as the internal standard), the HDL proteins were incubated overnight at 37°C with 20:1 (w/w, protein/enzyme) of sequencing-grade modified trypsin. Digestion was halted by acidifying the reaction mixture (to pH 2–3) with trifluoroacetic acid, and the digested samples were dried and stored at −80°C until MS analysis.

### LC-ESI-MS/MS analysis of HDL-associated proteins by parallel reaction monitoring

To quantitatively measure the relative levels of HDL proteins, we used targeted proteomics with isotope-dilution parallel reaction monitoring (PRM) as previously described (22). Briefly, LC-ESI-MS/MS analyses were performed in the positive ion mode with an ultrahigh-resolution accurate mass Orbitrap Fusion Tribrid Mass Spectrometer (Thermo Fisher Scientific, San Jose, CA) coupled to a nanoACQUITY UPLC (Waters, Milford, MA). A multistep gradient of 0.1% formic acid in water (solvent A) and 0.1% formic acid in acetonitrile (solvent B) was used for the separation. HDL peptide digests (equivalent to 0.1 μg of protein) were desalted on a C-18 trap column (0.1 × 40 mm) at a flow rate of 2.5 μl/min for 8 min. They were then separated at a flow rate of 0.5 μl/min using a C-18 analytical column (0.1 × 200 mm). The trap and analytical columns were packed in-house with Magic C-18 reverse-phase resin (5 μm; 100 Å; Michrom Bioresources). The columns were kept at room temperature, and the peptides were separated using a multistep gradient as follows: 1–7% solvent B for 1 min; 7–25% solvent B for 24 min; 25–35% solvent B for 6 min; and 35–80% solvent B for 5 min. The column was subsequently washed for 3 min at 80% B and re-equilibrated at 99% A for 12 min. The mass spectrometer was operated in the data-independent acquisition PRM mode.

Initially, the potential peptides for each protein were selected from the detected peptides by shotgun analysis and from our previous studies (18, 19). At least two peptides from one protein were then tested by PRM test runs, and finally, two or more peptides were selected for 21 proteins and one peptide for 10 proteins (supplemental Table S2). All peptides selected for one protein were unique to that protein. Because oxidation of methionine residues might affect the quantification, we avoided methionine containing peptides.

### Quantifying HDL proteins with ^15^N-labeled APOA1

The relative level of a protein in HDL was quantified as previously reported (18, 19). Briefly, the targeted PRM MS data of peptides of each HDL protein (supplemental Table S2) were analyzed using Skyline (version 20.2.0.286), an open-source program (23). An equal amount of ^15^N-labeled APOA1 was added to HDL isolated from each subject prior to digestion as an internal standard. Based on the journal guidance from the *Molecular & Cellular Proteomics*, our targeted PRM analyses belong to tier level 3. The peak areas of all the transitions of a peptide detected by PRM analysis were summed to get the total peak area for the peptide, but the transitions with interferences were deleted. To normalize the peak area of a peptide, the total peak area of all selected transitions of the peptide was divided by the peak area of each of the four ^15^N-labeled peptides from ^15^N-APOA1 (supplemental Table S2), and the average of the four ratios was used for quantification. To calculate the relative levels of the peptide between control and incident CVD groups, we set the average ratio of the peptide in control subjects as an arbitrary unit of one. If two or more peptides were quantified for a protein, the relative levels of all peptides from the protein were averaged to obtain the relative level of that protein in HDL.

### Statistical analysis

Baseline demographic and clinical characteristics were compared using a two-tailed unpaired Student's *t-*test for normally distributed data or the Mann-Whitney nonparametric *U* test for abnormally distributed data. We used the Shapiro-Wilk tests to assess the normality of distribution. Categorical variables were compared using the Chi-square test to compare frequencies between groups. To account for multiple comparison testing of HDL proteins, we first used the Benjamini-Hochberg method with a 10% false discovery rate (i.e., based on corrected or adjusted *P* values [*q* values], only proteins with a *q* value of <0.10 were initially considered significant). The false discovery rate is only the threshold for selecting candidate proteins for further analysis. And then, univariate and multivariate models were built for the significant proteins using logistic regression analysis with incident CVD as a dependent variable. We tested associations of HDL proteins with incident CVD using one-to-one matched multinomial logistic regression analysis, and the results were expressed in odds ratios (ORs) with a 95% confidence interval. The logistic regression models were adjusted for potential clinical confounders (including age, hypertension, present smoker, statin use, body mass index, and eGFR; gender and diabetic status were matched one to one) (model 1). And then, the models were further adjusted for levels of lipids (including HDL-C, LDL-C, and triglycerides) (model 2) or together with proteinuria and C-reactive protein (CRP) (model 3). ORs were reported per 1 SD increment of differences of HDL protein levels between matched subjects. In addition, we compared the associations between HDL proteins and incident CVD in stratified subgroups to assess any differences in ORs across categories of subject characteristics, including diabetic status (yes vs. no), gender (female vs. male), age (<55 vs. >55, i.e., the median value, same as below for continuous variables), hypertension (yes vs. no), eGFR (<45 vs. >45 ml/min/1.73 m^2^), HDL-C (<45 vs. >45 mg/dl), CRP (<0.38 vs. >0.38 mg/dl), and urinary protein to creatinine ratio (<1.25 vs. >1.25). Two-sided *P* values <0.05 were considered statistically significant. All statistical analyses were performed using SPSS (Windows version 19; SPSS, Inc, Chicago, IL) or R (a free software environment for statistical computing and graphics; version 4.0.5).

## Results

The clinical characteristics of the 92 CKD subjects (46 controls without CVD outcomes and 46 with incident CVD) are listed in Table 1. We matched the two groups (control and incident CVD) by diabetic status and gender. We also tried to roughly match age (within 10 years for matched pairs), percentages of whites, and CKD stages (or levels of eGFR) for the matched pairs. We did not match the other parameters in the table, but the two groups had similar levels of HDL-C, LDL-C, total cholesterol, triglycerides, CRP, serum creatinine, systolic blood pressure, and diastolic blood pressure. The incident CVD group had significantly higher urinary protein to creatinine ratios.

**Fig. 1:**
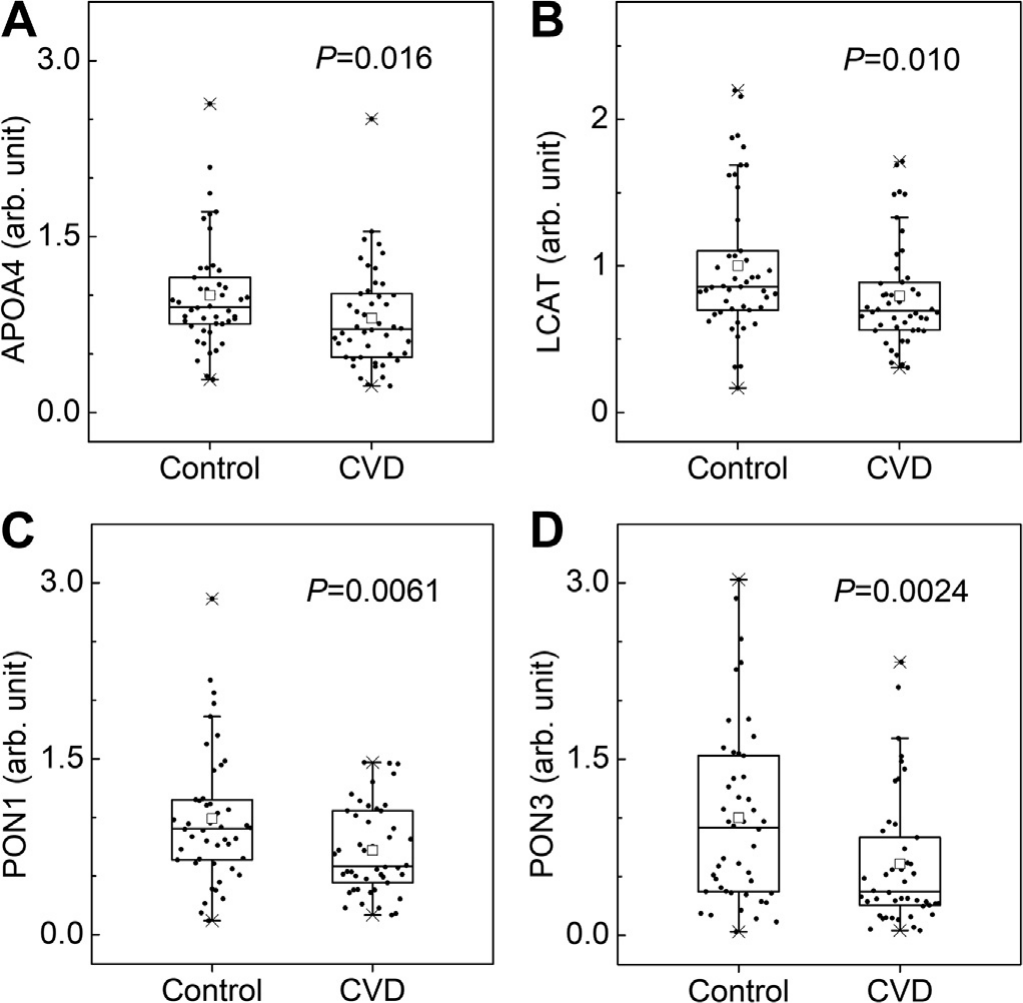
PRM analysis of HDL proteins in CKD patients with or without incident CVD. A: APOA4, (B) LCAT, (C) PON1, and (D) PON3. HDL was isolated, and HDL proteins were analyzed as described in Materials and methods section. The average levels of proteins in HDL isolated from the control group were set at an arbitrary unit of one. The box plots show the distribution of the data of HDL proteins (median and interquartile ranges), whereas the dots represent individual data points. Crosses (x) represent 99% and 1% levels. The small squares within the boxes represent mean levels. *P* values are from a Mann-Whitney *U* test.

To assess whether specific HDL proteins predict incident CVD in CKD patients, we used tandem MS analysis with targeted PRM and isotope dilution to quantify relative levels of 31 proteins in the HDLs from the two groups of subjects. We selected these 31 proteins based on our previous studies, which include the proteins implicated in CKD and CVD (18, 19) and most of the apolipoproteins in HDL (supplemental Table S2). In this study, we detected all 31 proteins in HDL isolated by ultracentrifugation from each of the 92 subjects. Differences in protein expression were initially evaluated without adjustment for clinical characteristics but with multiple comparisons (Benjamin-Hochberg-adjusted *P* value < 0.10). That analysis revealed that six proteins were differentially expressed in subjects with incident CVD events as compared with control subjects (Table 2 and supplemental Table S3). The six proteins were APOA1, APOA4, APOC3 (apolipoprotein C-III), LCAT, PON1, and PON3. Levels of all these proteins—except for APOC3—were significantly lower in HDL isolated from subjects with incident CVD events than in control HDL. Levels of four HDL proteins—APOA4, LCAT, PON1, PON3—in control and CVD groups are shown in Fig. 1. These observations demonstrate that changes in the protein composition of HDLs associate with incident CVD in CKD patients.

**Table 2:** PRM analysis of HDL proteins

Protein	Control	CVD	*P*
PON3	1 ± 0.76	0.59 ± 0.55	0.0024
PON1	1 ± 0.56	0.71 ± 0.36	0.0061
APOC3	1 ± 0.45	1.21 ± 0.47	0.0097
LCAT	1 ± 0.49	0.79 ± 0.35	0.010
APOA4	1 ± 0.46	0.80 ± 0.43	0.016
APOA1	1 ± 0.28	0.86 ± 0.24	0.018

HDL was isolated from plasma of 46 CKD subjects without CVD events (control) and 46 CKD subjects with CVD events (CVD). Following the digestion of HDL with trypsin, the tryptic digests of HDL proteins were analyzed by isotope dilution-targeted MS/MS with PRM. The average levels of proteins in HDL isolated from the control group were set as an arbitrary unit of one. Data shown are mean ± SD. Because the levels of proteins in HDL are not normally distributed even after log transformation, *P* values were obtained by a Mann-Whitney nonparametric test. HDL proteins that differed significantly between the control and CVD groups when the Benjamini-Hochberg false discovery rate-adjusted *P* values were controlled at 10% are shown.

We previously demonstrated that a cluster of proteins in HDL is dramatically increased in end-stage renal disease (ESRD) patients on hemodialysis (HD) (18). We confirmed the observation in the current study. Thus, levels of α-1-microglobulin/bikunin precursor, beta-2-microglobulin, complement factor D, cystatin-C, and retinol-binding protein 4 in HDL were strongly correlated with levels of eGFR (supplemental Table S4) and steadily increased as CKD became more severe (supplemental Fig. S1). Considering the low numbers of subjects in CKD stages 1 and 2 and stages 4 and 5, we combined the first two stages and the last two stages to form three new groups: mild (CKD1 and CKD2), moderate (CKD3), and severe (CKD4 and CKD5). However, there was no association between levels of those proteins and incident CVD at any stage of CKD (supplemental Fig. S1). In contrast, of the six proteins that were differentially expressed in the incident CVD group compared with the control group, only the levels of APOA4 in HDL correlated with eGFR (supplemental Table S4) and were steadily increased as CKD became more severe (supplemental Fig. S2). It is noteworthy that four of the six proteins (APOA1, APOA4, APOC3, and PON3) were only significantly associated with incident CVD at stages 1–2 but not at higher stages (supplemental Fig. S2), probably because of the small number of subjects at each stage and the dramatic changes in HDL proteome in higher CKD stages.

**Fig. 2:**
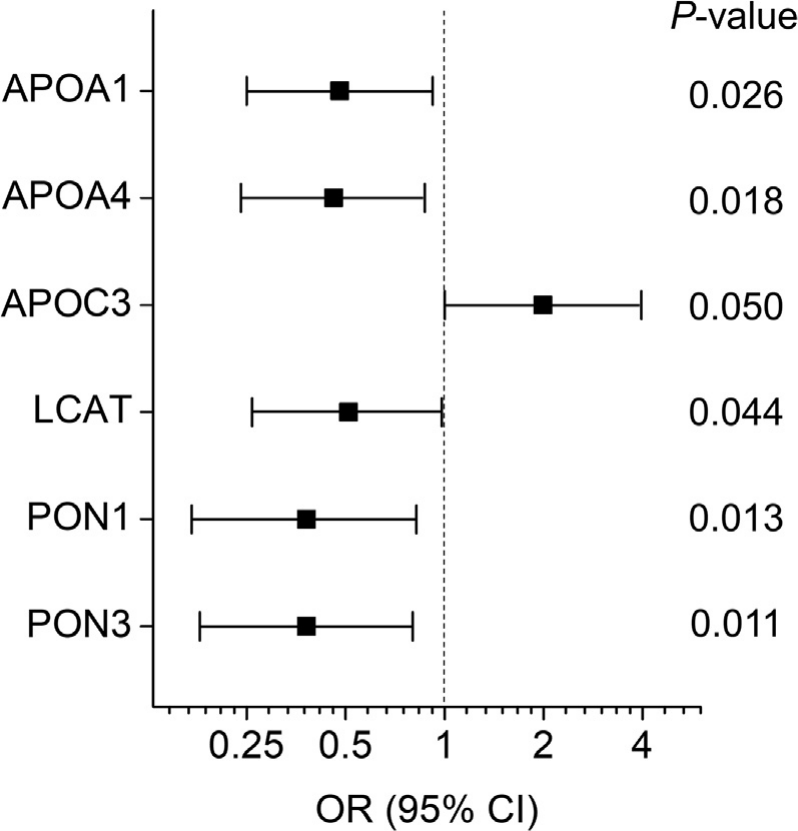
ORs of HDL proteins for incident CVD. Unadjusted ORs, 95% confidence interval (CI), and *P* values were obtained from a one-to-one matched multinomial logistic regression analysis. ORs are per SD increase in differences of levels of HDL proteins between matched subjects.

We used one-to-one matched multinomial logistic regression analysis to assess the association of HDL proteins with incident CVD. First, we obtained unadjusted ORs for the six significantly altered HDL proteins and HDL-C in predicting incident CVD. As shown in Fig. 2 and supplemental Table S5, the differences in all six proteins in the one-to-one matched subjects were associated significantly with incident CVD. The differences in levels of APOC3 were associated positively with incident CVD, whereas the differences in the other five proteins were associated inversely with incident CVD. This analysis also revealed that the differences in levels of HDL-C did not associate with incident CVD.

**Fig. 3:**
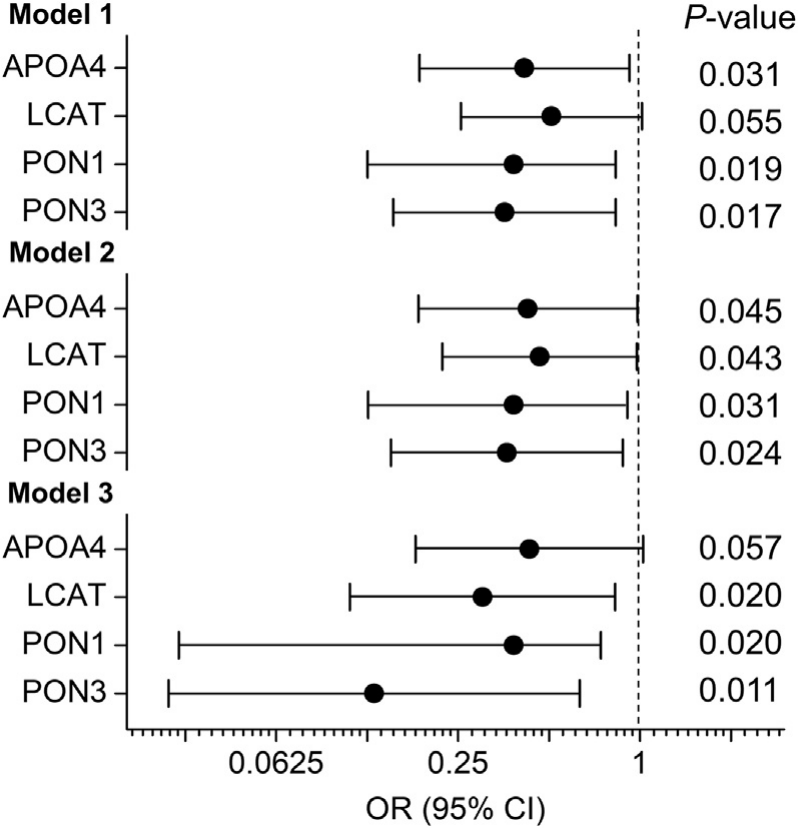
ORs of HDL proteins for incident CVD after adjustment for potential confounders. ORs, 95% confidence interval (CI), and *P* values were obtained from a one-to-one matched multinomial logistic regression analysis. Model 1: ORs after adjustment for clinical characteristics, including age, hypertension status, present smoker, statin use, BMI, and eGFR; model 2: ORs after adjustment for clinical characteristics (see model 1) and levels of lipids (HDL-C, LDL-C, and triglycerides); model 3: ORs after adjustment for clinical characteristics (see model 1), levels of lipids (see model 2), proteinuria, and CRP. ORs are per SD increase in differences in levels of HDL proteins between matched subjects. BMI, body mass index.

Next, we adjusted the models for clinical confounders, including age, hypertension, present smoker, statin use, body mass index, and eGFR. The subjects were matched one to one by diabetic status and gender. Therefore, the diabetic status and gender were left out of the models. The differences in levels of five of the six proteins (except LCAT) remained significantly associated with incident CVD after adjustment for clinical confounders (supplemental Table S6 and Fig. 3, model 1). We then added levels of lipids, including HDL-C, LDL-C, and triglycerides, into model 1. The multivariate logistic regression analyses demonstrated that the associations between the differences of levels of APOA4, LCAT, PON1, and PON3 in HDL and incident CVD were significant (Fig. 3, model 2 and supplemental Table S7). The other two proteins (APOA1 and APOC3) did not associate with incident CVD after adjustment for clinical confounders and lipid levels. Finally, when we added proteinuria and CRP into model 2, differences in three proteins, LCAT, PON1, and PON3, maintained significant associations with incident CVD (Fig. 3, model 3 and supplemental Table S8). Our observations demonstrated that two HDL proteins, PON1 and PON3, were significantly associated with incident CVD in all the models adjusted for clinical characteristics, lipid levels, and proteinuria and CRP (Fig. 3, models 1–3). Though LCAT was not significant when the model was only adjusted for clinical characteristics (model 1), it was significantly associated with incident CVD in more completely adjusted models (models 2 and 3). Similarly, while remaining significant in models 1 and 2, APOA4 was not significant in the final model when proteinuria and CRP levels were also added into the model (model 3).

**Fig. 4:**
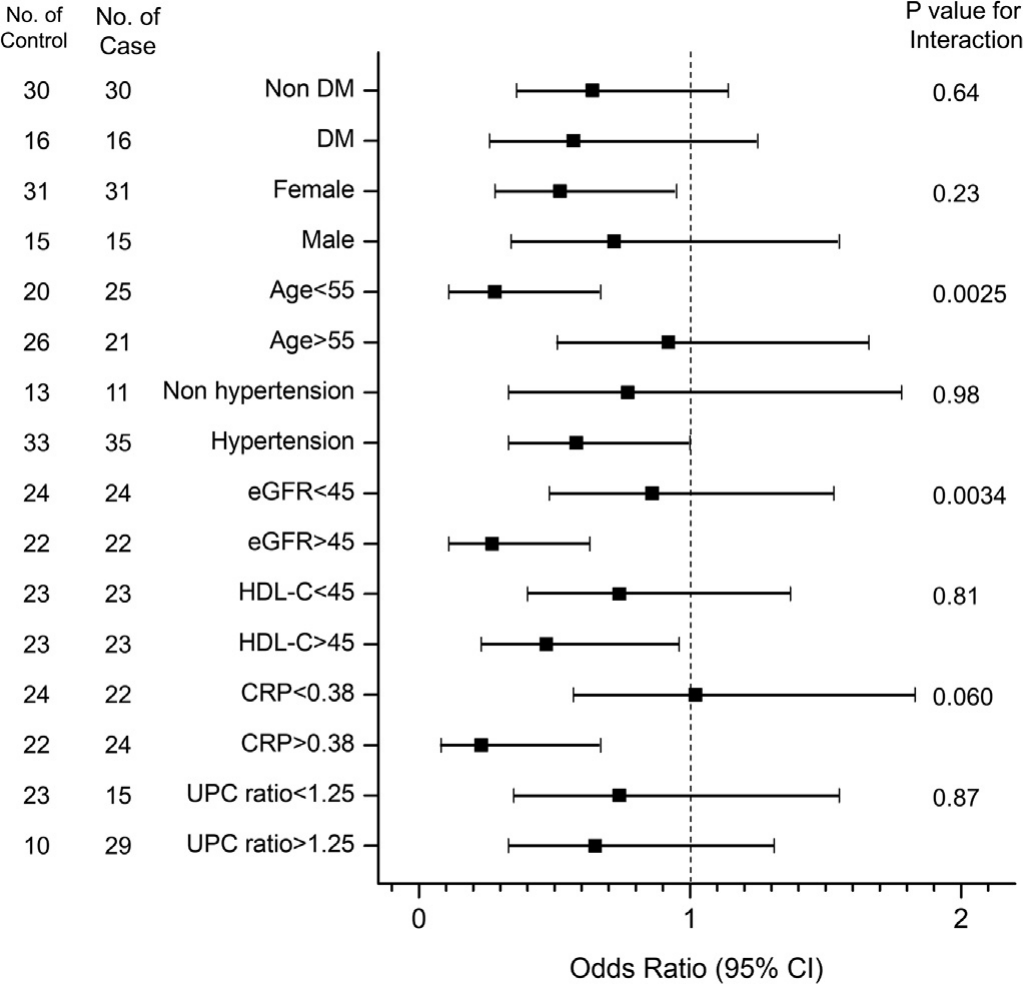
OR of APOA4 by stratification for incident CVD. Unadjusted ORs of APOA4 in HDL predicting incident CVD are obtained from a logistic regression analysis in stratified subgroups to assess any differences in ORs across categories of subject characteristics, including diabetic status (yes vs. no), gender (female vs. male), age (<55 vs. >55), hypertension (yes vs. no), eGFR (<45 vs. >45 ml/min/1.73 m^2^), HDL-C (<45 vs. >45 mg/dl), CRP (<0.38 vs. >0.38 mg/dl), and UPC ratio (<1.25 vs. >1.25). *P* values for interaction were obtained from a multivariable logistic regression analysis with an interaction term (APOA4 times one clinical characteristic) added into the model. UPC ratio, urinary protein to creatinine ratio.

To assess any differences across categories of the subjects' characteristics, we compared the associations between four HDL proteins (APOA4, PON1, PON3, and LCAT) and incident CVD by logistic regression analysis in stratified subgroups (Fig. 4 and supplemental Figs. S3–S5). Stratifying the subjects into subgroups did not change the inverse association of the four HDL proteins with incident CVD but did affect the significance levels. Thus, levels of all four HDL proteins were associated inversely and significantly with incident CVD events only in patients who were younger than 55 years. For APOA4, PON1, and PON3, they were associated inversely and significantly with incident CVD only in patients who were female, hypertensive, and had HDL-C above 45 mg/dl. In contrast, CRP and eGFR affected the associations differently. While levels of APOA4 were associated with incident CVD only in subjects with CRP >0.38 mg/dl, levels of PON1 were associated with incident CVD only in subjects with CRP <0.38 mg/dl. On the other hand, while levels of APOA4 and PON1 were significantly associated with incident CVD only in subjects with eGFR greater than 45 ml/min/1.73 m^2^, PON3 and LCAT were significant only in subjects with eGFR less than 45 ml/min/1.73 m^2^.

Although stratification did not change the inverse relationship between levels of APOA4 and incident CVD, there were significant interactions between levels of APOA4 and age and eGFR and nearly significant for CRP (Fig. 4). Thus, the inverse association between levels of APOA4 and incident CVD were much stronger in subjects who were younger than 55 years, had eGFR greater than 45 ml/min/1.73 m^2^, and CRP greater than 0.38 mg/dl. It is noteworthy that there were no significant interactions between other three proteins (PON1, PON3, and LCAT) and any of the clinical characteristics.

## Discussion

Our study is the first to quantify changes in the HDL proteome and their associations with incident CVD in CKD subjects. We used a matched prospective case-control design, targeted MS/MS analysis, and one-to-one matched multinomial logistic regression analysis to demonstrate that six HDL proteins (APOA1, APOA4, APOC3, LCAT, PON1, and PON3) were associated with incident CVD in CKD patients. In our fully adjusted models (including clinical confounders, lipid levels, CRP, and proteinuria), lower levels of three HDL proteins, LCAT, PON1, and PON3, were significantly associated with incident CVD in CKD subjects. On the other hand, reduced levels of APOA4 in HDL remained to have a significant association with incident CVD after adjustment for clinical confounders and lipid levels but lost significance in the final model that included CRP and proteinuria. In contrast, the association between levels of LCAT in HDL and incident CVD was not significant when we only adjusted the model for clinical confounders, but the association became significant when we further adjusted the model for lipid levels and included CRP and proteinuria.

Traditionally, HDL-C is inversely associated with the risk of future cardiovascular events. Low HDL-C is associated with poor kidney function and progression of renal disease (20). It is unclear, however, whether low HDL-C contributes to increased CVD risk in patients with CKD. Interestingly, a recent study of CKD patients on HD revealed a U-shaped association of increased risk with total and CVD mortality in patients when HDL-C concentrations were <30 and >60 mg/dl (24). In the current study, the two groups had similar HDL-C levels, and HDL-C levels did not associate with eGFR (the kidney function) or incident CVD in CKD patients.

Several studies, including ours, have characterized the altered HDL proteome with decreasing renal function (18, 25, 26), but it is unknown whether the HDL proteome in CKD patients is associated with incident CVD events. In the current study, low levels of PON1 and PON3 in HDL were associated with incident CVD in our CKD cohort after adjustment for various potential CVD risk factors. These two members of the *PON* gene family are carried exclusively by circulating HDL, though the concentration of PON3 in serum is about two orders of magnitude lower than that of PON1 (27). On an atherogenic diet, mice deficient in PON1 develop atherosclerosis faster than control mice. Conversely, overexpression of PON1 protects mice from atherosclerosis, suggesting that PON1 is strongly antiatherogenic in vivo (28, 29). Its antiatherogenic activities are attributed to its antioxidative and anti-inflammatory properties (30). Importantly, levels of PON1 activity in HDL associate negatively with CVD in multiple human studies (31, 32).

Both PON1 activity and mass are significantly lower in CKD patients with or without HD than in healthy control subjects (33). It is noteworthy that the lower enzymatic activity of PON1 in CKD patients is independent of changes in HDL-C. Moreover, it does not associate with the L55M and Q192R allelic polymorphisms that associate with atherosclerosis (34). However, few prospective studies of the prognostic value of PON1 mass and/or activity for predicting incident CVD events in CKD patients exist. In one observational study of outpatient HD population, low PON1 concentration is associated with cardiovascular mortality and all-cause mortality even after correction for known risk factors for CVD or mortality in HD patients. This suggests that a low concentration of PON1 might be an independent predictor of cardiovascular mortality in maintenance HD patients (35). In another prospective study of CKD patients initially undergoing dialysis, decreased serum PON1 activity predicted a higher risk of incident cardiovascular events after adjustment for established clinical and biochemical risk factors (36). However, the CARE FOR HOMe study with nondialysis CKD patients demonstrated that the quantity, composition, and function of HDL, including PON activity, do not independently predict cardiovascular outcomes after adjustment for traditional cardiovascular and renal risk factors (37). In contrast, in patients with maintenance HD, PON activity had a stronger association with 12-month all-cause mortality than HDL-C and APOA1 in fully adjusted models (38).

Like PON1, PON3 appears to have both antioxidant and antiatherogenic functions in animal and in vitro studies (39). PON3-deficient mice had significantly larger atherosclerotic lesions (40), and overexpression of human PON3 significantly reduced diet-induced atherosclerotic lesions in several mouse models (41), indicating that PON3 is antiatherogenic in hypercholesterolemic mice. Serum PON3 is significantly higher in patients with peripheral artery disease or coronary artery disease than control subjects (42) but significantly lower in uremic subjects on HD (18). Importantly, a low concentration of PON3 in HDL associates with atherosclerosis in patients with type 1 diabetes and autoimmune disease (19, 43). However, the value of PON3 for predicting future CVD events has not been studied in either CKD patients or the general population.

In the current study, we demonstrated that low levels of APOA4 in HDL were significantly and inversely associated with incident CVD in our CKD cohort after adjustment for traditional clinical cofounders and lipid levels. A primary function of APOA4 is to accept cholesterol from macrophages during reverse cholesterol transport via the ABCA1 pathway, and its effect is comparable to that of APOA1 and APOE at physiologically relevant concentrations (44). Like APOA1, APOA4 can activate LCAT and cholesteryl ester transfer protein (45, 46). In addition, it has anti-inflammatory and antioxidative properties (47, 48). Importantly, overexpression of APOA4 protected against atherosclerosis in multiple studies with different strains of mice, likely by a mechanism that does not increase the concentration of HDL-C, suggesting that APOA4 by itself is antiatherogenic (47, 49).

In clinical studies, a low concentration of plasma APOA4 is associated with coronary artery disease, and this association is independent of HDL-C and triglyceride concentrations (50). Circulating APOA4 binds to LDL and HDL, but a large portion of APOA4 in plasma is lipid free. In order to determine whether plasma APOA4 also predicts incident CVD in CKD patients, we measured the levels of APOA4 in plasma for the 92 samples. As shown in supplemental Fig. S6, the levels of APOA4 in plasma were lower in patients with CVD events (1 ± 0.58 vs. 0.91 ± 0.48, control vs. CVD), but the difference between the control group and the CVD group was not significant (*P* = 0.47). In contrast, we demonstrated that low levels of APOA4 in HDL predict incident CVD events in CKD patients. The small sample size might be an important factor for the different results we observed between the APOA4 levels in HDL and plasma. These observations raise the possibility that HDL protein composition might be an earlier marker for the development of CVD than protein changes in plasma, and therefore, it might be important to measure HDL proteomics and plasma proteomics independently.

On the other hand, increased APOA4 concentrations are linked to CKD progression and ESRD, independently of CKD risk factors (51, 52). However, serum APOA4 concentrations are negatively associated with the presence and development of atherosclerosis in both HD (ESRD) patients and patients with mild to moderate renal failure (51, 53). Consistent with the aforementioned observations, levels of APOA4 in HDL steadily increased with the progression of CKD in the current study. It is noteworthy that there were strong interactions between HDL's APOA4 content and age, eGFR, and also CRP for predicting incident CVD in CKD patients. The inverse associations between APOA4 and incident CVD were more robust in younger subjects and milder CKD. Our observations suggest that APOA4 is a better predictor of future CVD events when kidney function is not severely impaired, probably because the levels of APOA4 in HDL increase in later stages of CKD. Moreover, APOA4 was significantly associated with incident CVD only in subjects with CRP levels greater than 0.38 mg/dl. The interaction between APOA4 and CRP levels, overfitting with 11 variables, and small sample size might be the reasons APOA4 lost significance in a model that includes CRP in addition to clinical confounders and proteinuria.

LCAT is an enzyme that catalyzes the esterification of free cholesterol on HDL, which is responsible for HDL maturation. However, its role in the pathogenesis of atherosclerosis is still debated, and the results from both animal and human studies remain contradictory (54). Renal disease represents the major cause of morbidity and mortality in familial LCAT deficiency cases (55), but it is unknown whether the levels of LCAT in HDL associate with the development of CVD in CKD patients. In our previous study, we found that LCAT in HDL is negatively and significantly associated with coronary artery calcification in the model adjusted for multiple clinically relevant covariates, but the association loses significance when the model was further adjusted for lipid levels (19). In the current study, we observed lower levels of LCAT in HDL isolated from CKD patients with incident CVD events compared with control CKD subjects. Importantly, the levels of LCAT in HDL were significantly associated with incident CVD in a model adjusted for both clinical confounders and lipid levels.

Strengths of our study include a well-validated targeted MS/MS approach for quantifying HDL proteins, the matched and similar clinical characteristics of the incident CVD cases and control subjects at different stages of CKD, and a stringent statistical approach using one-to-one matched multinomial logistic regression analysis. A limitation of our approach is that association does not prove causality. It will be essential to confirm and extend our findings with larger cohorts of CKD subjects with longer follow-up as our study was underpowered to detect changes in specific HDL proteins. Our small sample size and the subsequent decrease in power weakened the adjusted models with APOA4 and LCAT and limited us from providing important insights into the physiology behind the observed associations between incident CVD events and specific HDL proteins. In future studies, we will need to investigate the molecular mechanisms, whereby APOA4, LCAT, PON1, and PON3 protect against atherosclerosis in CKD and if improving their levels in HDL will reverse CKD atherosclerosis in model systems.

In conclusion, we demonstrated that levels of four proteins in HDL—APOA4, LCAT, PON1, and PON3—were inversely associated with incident CVD events in patients with CKD. Importantly, PON1 and PON3 were significant in all three adjusted models, indicating that the associations were independent of all clinical characteristics and CVD risk factors, including proteinuria and CRP. Given their protective functions against atherosclerosis and their yet unidentified roles in kidney disease, APOA4, LCAT, PON1, and PON3 may represent therapeutic targets for CVD in CKD patients. Our observations support the proposal that the protein cargo of HDL can serve as a marker—and perhaps mediator—of CVD risk in CKD patients.

## Data availability

The data supporting this study are available in the article, the supplemental data, or available from the corresponding author upon reasonable request. The raw MS data have been deposited at the Panorama server (https://panoramaweb.org/hdlcprobe.url).

## Supplemental data

This article contains supplemental data.

## Conflict of interest

The authors declare that they have no conflicts of interest with the contents of this article.
