# Supplementary material for: Altered HDL proteome predicts incident CVD in chronic kidney disease patients
Source: J Lipid Res. 2021 Oct 9;62:100135. doi: 10.1016/j.jlr.2021.100135 (PMC8566900; doi:10.1016/j.jlr.2021.100135)
Supplement: Supplemental Figures S1–S6 [file mmc1.pdf]

Supplemental Figure 1

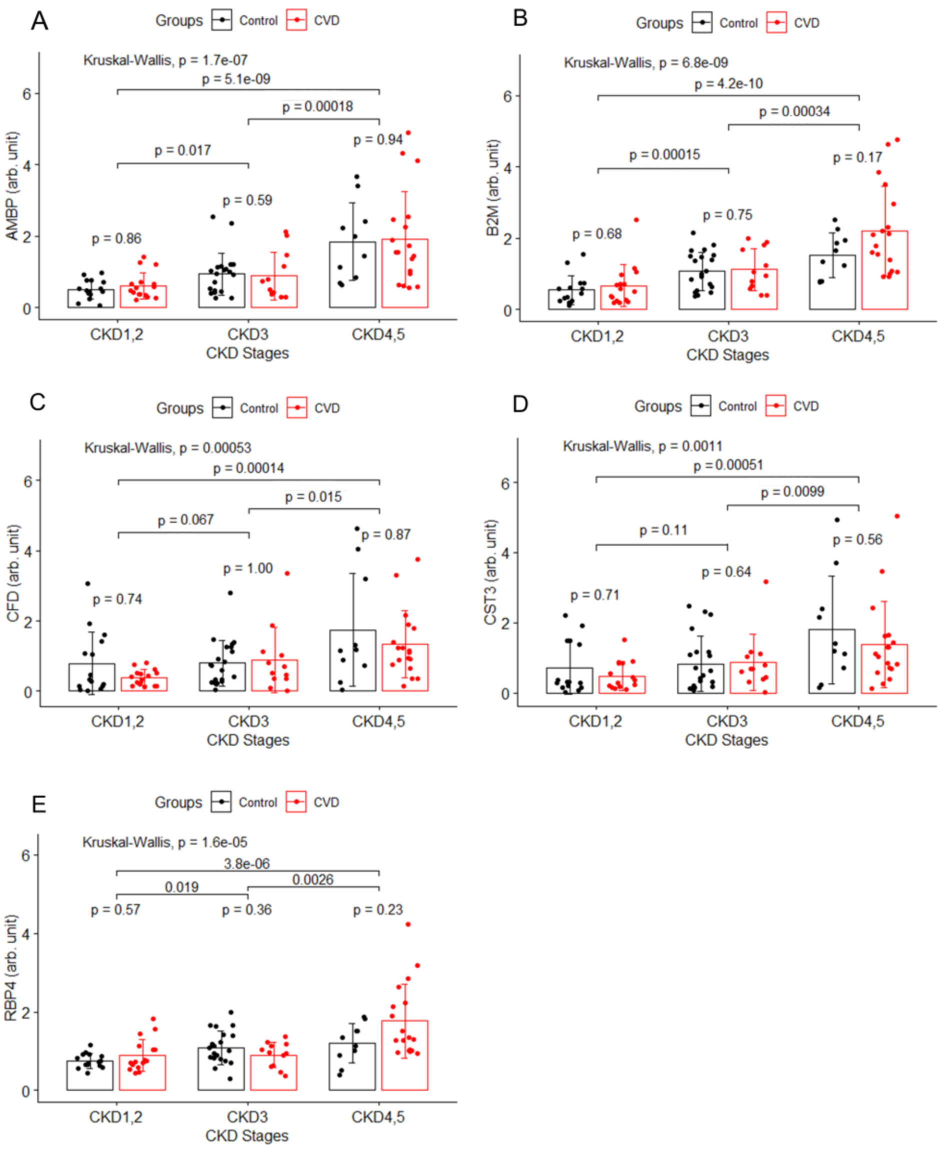

Supplemental Figure 1. Levels of HDL proteins enriched in ESRD by CKD stages and by CVD events. HDL was isolated from plasma of 46 CKD subjects without CVD events (control) and 46 CKD subjects with CVD events (CVD). Following the digestion of HDL with trypsin, the tryptic digests of HDL proteins were analyzed by isotope dilution targeted MS/MS with PRM. The average levels of proteins in HDL isolated from control group were set as an arbitrary unit of one. Data shown are mean ± SD for HDL proteins at different stages of CKD with CVD events (CVD) or without CVD events (control). CKD1,2 – combined chronic kidney disease stage 1 and 2 (control, N=15; CVD, N=16); CKD3 – chronic kidney disease stage 3 (control, N=21; CVD, N=12); CKD4,5 – combined chronic kidney disease stage 4 and 5 (control, N=10; CVD, N=18). AMBP, α-1-microglobulin/bikunin precursor; B2M, beta-2-microglobulin; CFD, complement factor D; CST3, cystatin-C; RBP4, retinol-binding protein 4.

Supplemental Figure 2

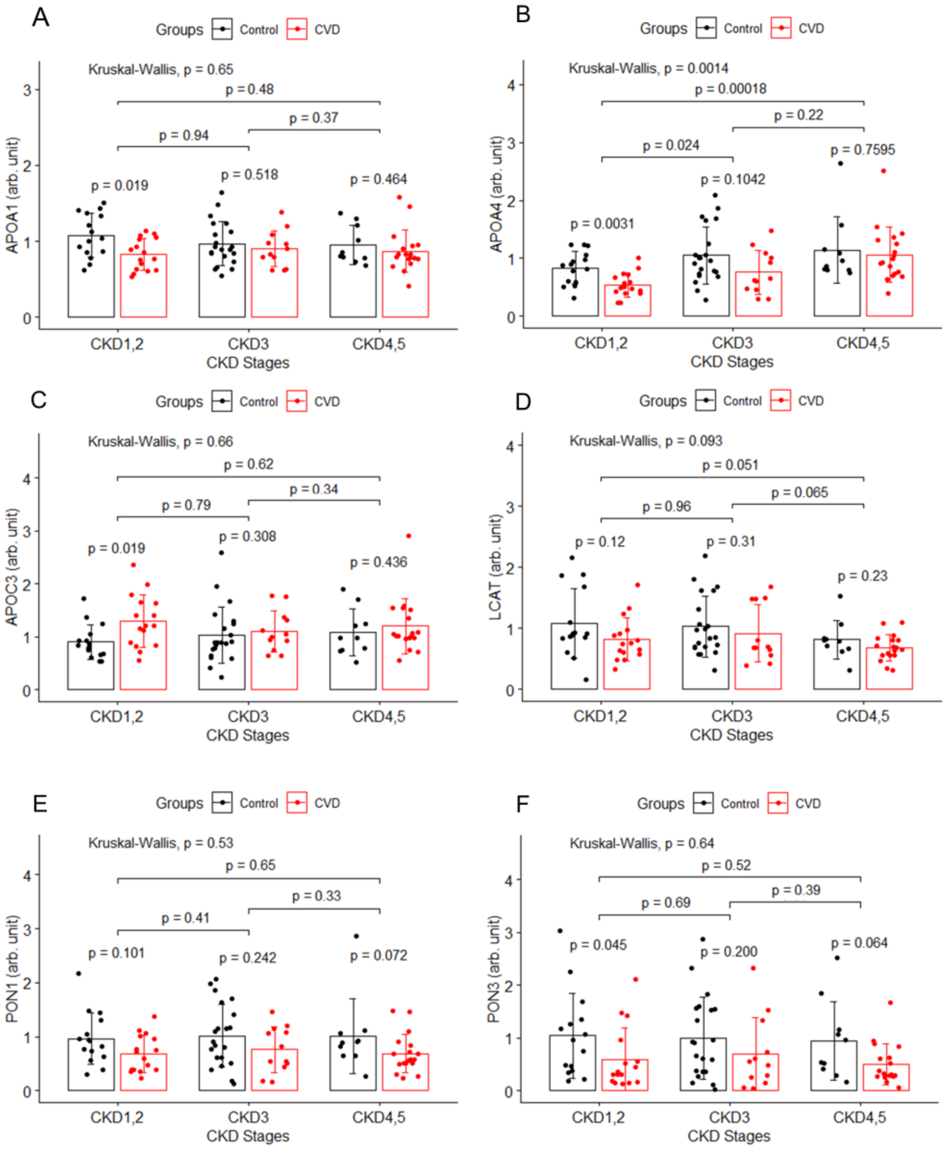

Supplemental Figure 2. Levels of primary affected HDL proteins by CKD stages and by CVD events. HDL was isolated from plasma of 46 CKD subjects without CVD events (control) and 46 CKD subjects with CVD events (CVD). Following the digestion of HDL with trypsin, the tryptic digests of HDL proteins were analyzed by isotope dilution targeted MS/MS with PRM. The average levels of proteins in HDL isolated from control group were set as an arbitrary unit of one. The box plots show the distribution of the data of HDL proteins (median, interquartile ranges) in different groups, while the dots represent individual data points. Crosses (x) represent 99% and 1% levels. The small squares within the boxes represent mean levels. CKD1,2 – combined chronic kidney disease stage 1 and 2 (control, N=15; CVD, N=16); CKD3 – chronic kidney disease stage 3 (control, N=21; CVD, N=12); CKD4,5 – combined chronic kidney disease stage 4 and 5 (control, N=10; CVD, N=18). APOA1, Apolipoprotein A-I; APOA4, Apolipoprotein A-IV, APOC3, Apolipoprotein C-III; LCAT, Lecithin:cholesterol acyltransferase; PON1, Paraoxonase/arylesterase 1; PON3, Paraoxonase/arylesterase 3.

Supplemental Figure 3

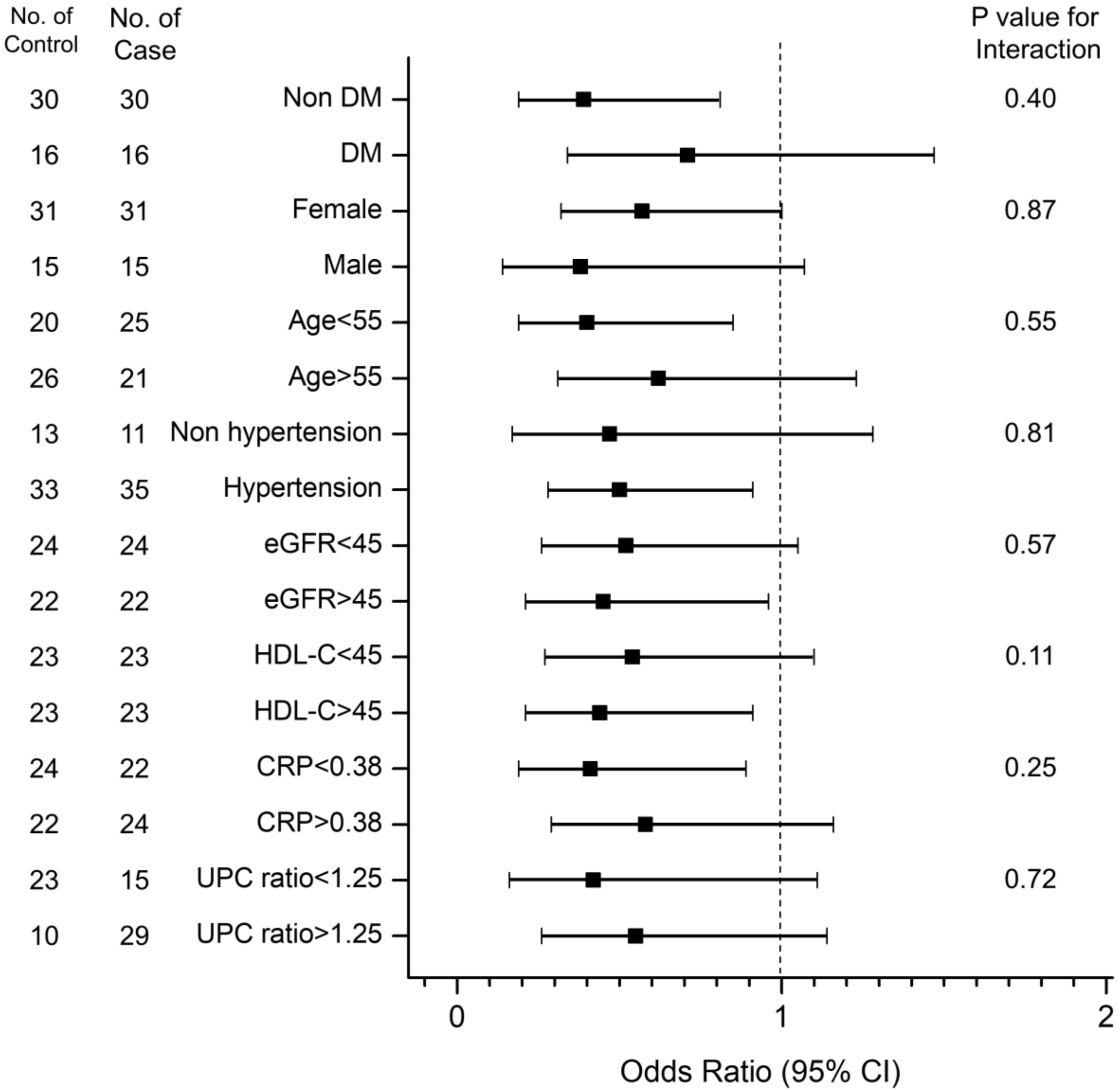

Supplemental Figure 3. Odds Ratio of PON1 by stratification for incident CVD. Unadjusted odds ratios of PON1 in HDL predicting incident CVD are obtained from a logistic regression analysis in stratified subgroups to assess any differences in ORs across categories of subject characteristics, including diabetic status (yes vs. no), gender (female vs. male), age (<55 vs. >55), hypertension (yes vs. no), eGFR (<45 vs. >45 mL/min/1.73 m^2), HDL-C (<45 vs. >45 mg/dL), CRP (<0.38 vs. >0.38 mg/dL), and UPC ratio (<1.25 vs. >1.25). P-values for interaction were obtained from a multivariable logistic regression analysis with an interaction term (PON1 times one clinical characteristic) added into the model. PON1, paraoxonase/arylesterase 1; eGFR: estimated glomerular filtration rate; HDL-C, HDL cholesterol; UPC ratio, urinary protein to creatinine ratio.

Supplemental Figure 4

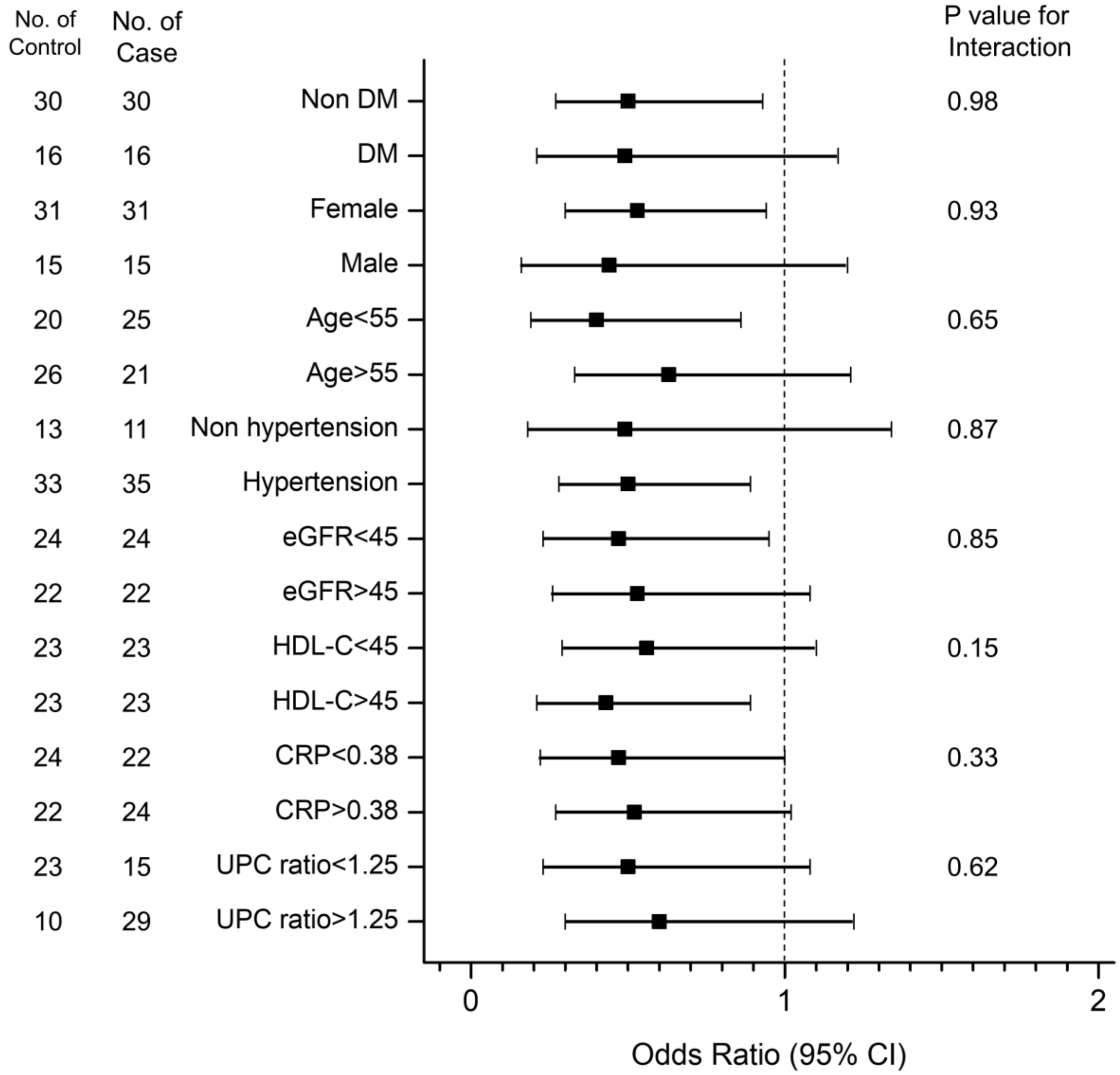

Supplemental Figure 4. Odds Ratio of PON3 by stratification for incident CVD. Unadjusted odds ratios of PON3 in HDL predicting incident CVD are obtained from a logistic regression analysis in stratified subgroups to assess any differences in ORs across categories of subject characteristics, including diabetic status (yes vs. no), gender (female vs. male), age (<55 vs. >55), hypertension (yes vs. no), eGFR (<45 vs. >45 mL/min/1.73 m^2), HDL-C (<45 vs. >45 mg/dL), CRP (<0.38 vs. >0.38 mg/dL), and UPC ratio (<1.25 vs. >1.25). P-values for interaction were obtained from a multivariable logistic regression analysis with an interaction term (PON3 times one clinical characteristic) added into the model. PON3, paraoxonase/arylesterase 3; eGFR: estimated glomerular filtration rate; HDL-C, HDL cholesterol; UPC ratio, urinary protein to creatinine ratio.

Supplemental Figure 5

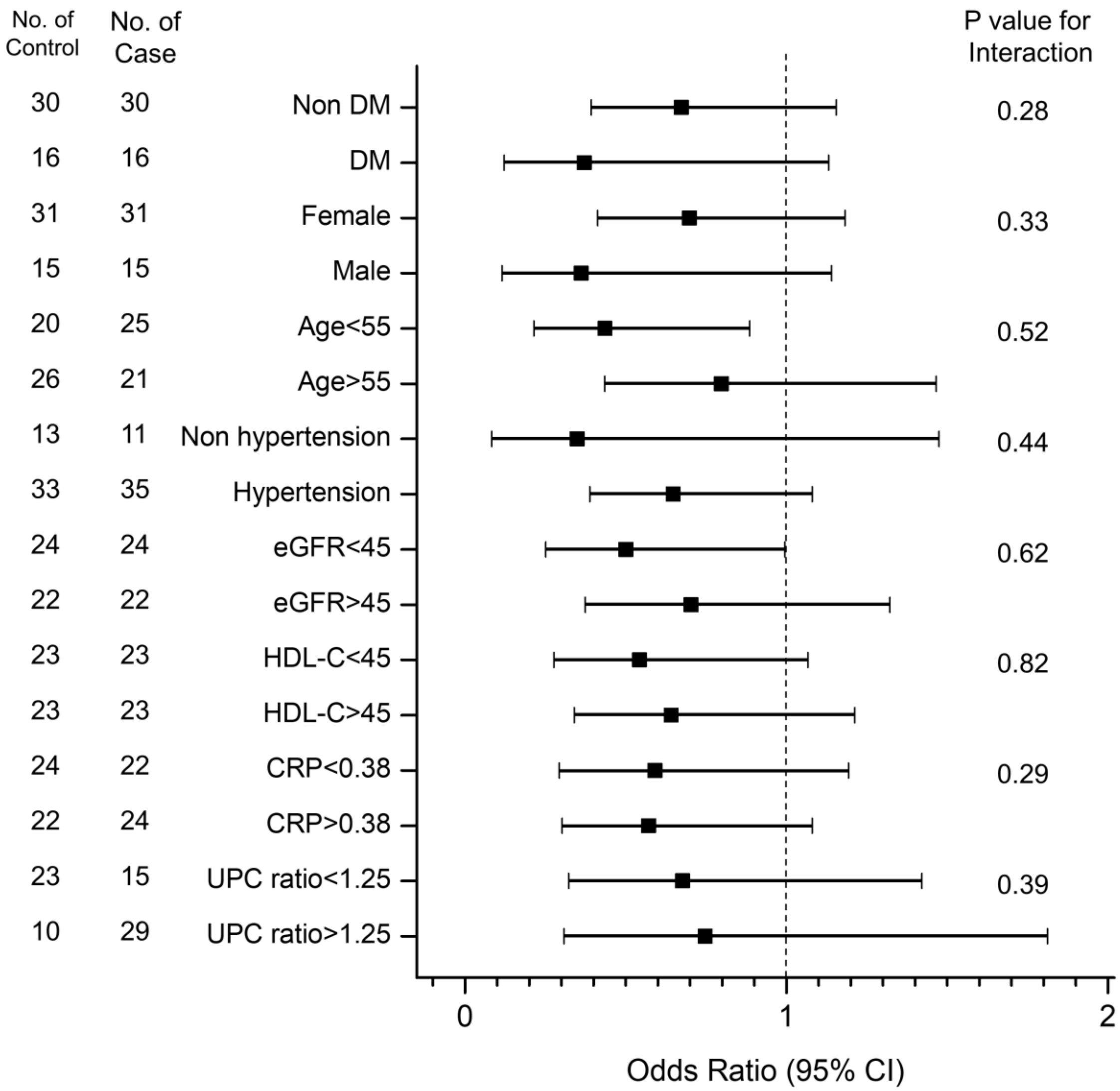

Supplemental Figure 5. Odds Ratio of LCAT by stratification for incident CVD. Unadjusted odds ratios of LCAT in HDL predicting incident CVD are obtained from a logistic regression analysis in stratified subgroups to assess any differences in ORs across categories of subject characteristics, including diabetic status (yes vs. no), gender (female vs. male), age (<55 vs. >55), hypertension (yes vs. no), eGFR (<45 vs. >45 mL/min/1.73 m^2), HDL-C (<45 vs. >45 mg/dL), CRP (<0.38 vs. >0.38 mg/dL), and UPC ratio (<1.25 vs. >1.25). P-values for interaction were obtained from a multivariable logistic regression analysis with an interaction term (LCAT times one clinical characteristic) added into the model. LCAT, Lecithin:cholesterol acyltransferase; eGFR: estimated glomerular filtration rate; HDL-C, HDL cholesterol; UPC ratio, urinary protein to creatinine ratio.

Supplemental Figure 6

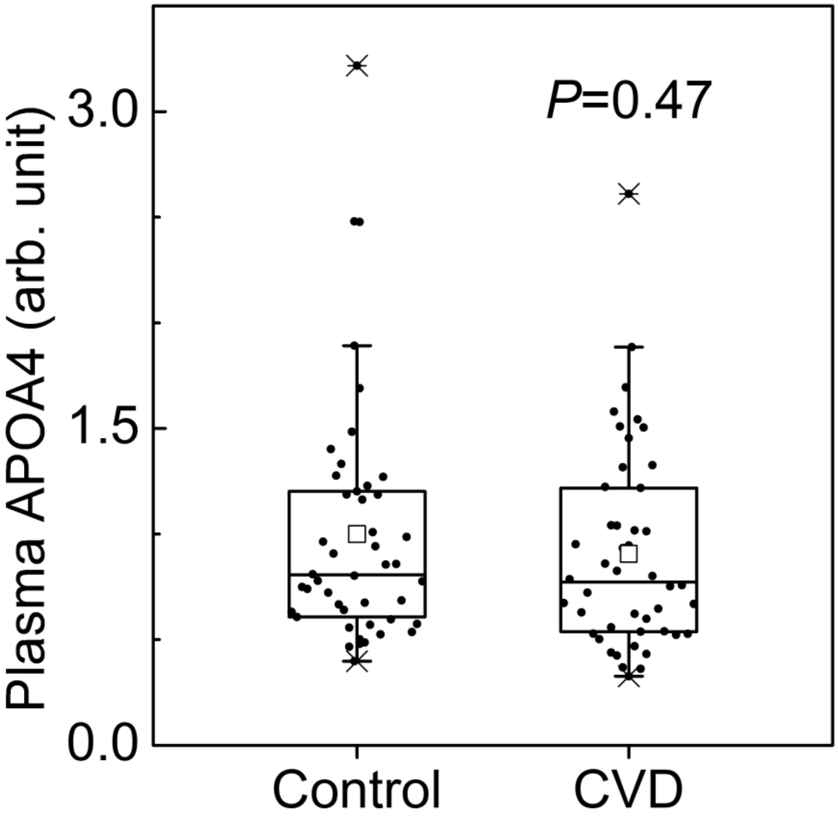

Supplemental Figure 6. PRM analysis of plasma APOA4 in CKD patients with or without incident CVD. The plasma samples of the 92 CKD patients were digested and the plasma proteins were analyzed and quantified as previous described (1). The average level of APOA4 in plasma of the control group was set at an arbitrary unit of one. The box plots show the distribution of the data of APOA4 (median, interquartile ranges), while the dots represent individual data points. Crosses (x) represent 99% and 1% levels. The small squares within the boxes represent mean levels. P-values are from a Mann-Whitney U test. APOA4, apolipoprotein A-IV.

1. Kanter, J. E., B. Shao, F. Kramer, S. Barnhart, M. Shimizu-Albergine, T. Vaisar, M. J. Graham, R. M. Crooke, C. R. Manuel, R. A. Haeusler, D. Mar, K. Bomsztyk, J. E. Hokanson, G. L. Kinney, J. K. Snell-Bergeon, J. W. Heinecke, and K. E. Bornfeldt. 2019. Increased apolipoprotein C3 drives cardiovascular risk in type 1 diabetes. *J Clin Invest* **130**: 4165-4179.
