# Supplementary material for: Altered HDL proteome predicts incident CVD in chronic kidney disease patients
Source: J Lipid Res. 2021 Oct 9;62:100135. doi: 10.1016/j.jlr.2021.100135 (PMC8566900; doi:10.1016/j.jlr.2021.100135)
Supplement: Supplemental Tables S1–S8 [file mmc2.pdf]

Supplemental Table 1. The Frequencies of CVD Endpoint Outcomes.

Endpoint Outcome	No. of Events
Arrhythmia	25
Chronic Atherovascular disease<br><br>(Atherosclerosis; angina; PVD; ischemic cardiomyopathy)	26
Heart failure	2
Acute MI	2
Acute Stroke/TIA	5
Thromboembolic Disease	2

CVD outcome is self-reported by patients and confirmed with electronic health record or by International Classification of Diseases-9 (ICD-9) code review at CPROBE and non-CPROBE sites.

Supplemental Table 2. Peptides Monitored for Each Protein by PRM Analysis.

Proteins	Description	Peptides	Precursor (m/z)	Charge State	RT (min)
AMBP	Alpha-1-Microglobulin/Bikunin Precursor	TVAACNLPIVR	607.3400	2	31.3
^{15}N-APOA1	^{15}N-labeled Apolipoprotein A-I	AKPALEDLR	513.2737	2	24.0
^{15}N-APOA1		DYVSQFEGSALGK	708.3160	2	37.6
^{15}N-APOA1		LHELQEK	454.2291	2	17.4
^{15}N-APOA1		VQPYLDDFQK	633.2948	2	32.6
APOA1	Apolipoprotein A-I	AKPALEDLR	506.7929	2	24.0
APOA1		DYVSQFEGSALGK	700.8380	2	37.6
APOA1		LHELQEK	448.7450	2	17.4
APOA1		VQPYLDDFQK	626.8141	2	32.6
APOA2	Apolipoprotein A-II	EQLTPLIK	471.2870	2	31.4
APOA2		SPELQAEAK	486.7540	2	19.6
APOA4	Apolipoprotein A-IV	LAPLAEDVR	492.2796	2	28.4
APOA4		LEPYADQLR	552.7878	2	28.4
APOA4		SELTQQLNALFQDK	817.9229	2	43.3
APOB	Apolipoprotein B	SPAFTDLHLR	578.8091	2	32.1
APOB		TEVIPPLIENR	640.8640	2	35.8

APOC1	Apolipoprotein C-I	EFGNTLEDK	526.7484	2	25.1
APOC1		EWFSETFQK	601.2798	2	37.3
APOC2	Apolipoprotein C-II	TAAQNLYEK	519.2670	2	21.5
APOC2		TYLPAVDEK	518.2715	2	27.8
APOC3	Apolipoprotein C-III	DALSSVQESQVAQQAR_2+	858.9292	2	28.5
APOC3		DALSSVQESQVAQQAR_3+	572.9552	3	28.5
APOC3		GWVTDGFSSLK	598.8009	2	39.0
APOC4	Apolipoprotein C-IV	AWFLESK	440.7320	2	37.1
APOD	Apolipoprotein D	NILTSNNIDVK	615.8380	2	31.2
APOE	Apolipoprotein E	AATVGSLAGQPLQER	749.4046	2	29.0
APOE		LGADMEDVCGR	611.7632	2	26.7
APOE2	Apolipoprotein E2	CLAVYQAGAR	554.7820	2	27.6
APOE4	Apolipoprotein E4	LGADMEDVR	503.2371	2	25.6
APOF	Apolipoprotein F	SGVQQLIQYYQDQK	849.4280	2	40.3
APOF		SYDLDPGAGSLEI	668.8170	2	42.3
APOL1	Apolipoprotein L-I	VAQELEEK	473.2480	2	19.2
APOL1		VTEPISAESGEQVER	815.8996	2	25.0

APOM	Apolipoprotein M	AFLLTPR	409.2502	2	33.9
APOM		NQEACELSNN	589.7408	2	20.6
B2M	Beta-2-Microglobulin	SNFLNCYVSGFHPSDIEVDLLK	852.0810	3	43.3
B2M		VNHVTLSQPK	561.8169	2	19.7
CAMP	Cathelicidin Antimicrobial Peptide	SSDANLYR	463.2220	2	20.7
CFD	Complement Factor D	LYDVLR	389.7265	2	31.0
CFD		RPDSLQHVLLPVLDR	586.6722	3	37.7
CLU	Clusterin	ELDESLQVAER	644.8230	2	28.8
CLU		LFDSDPITVTVPVEVSR	937.4989	2	41.4
CLU		VTTVASHTSDSDVPSGVTEVVVK	772.0639	3	29.8
CST3	Cystatin C	ALDFAVGEYNK	613.8060	2	34.5
GC	Vitamin D-binding protein	SCESNSPFPVHPGTAECCTK	755.6510	3	26.4
HPR	Haptoglobin-related protein	NPANPVQR	448.2409	2	18.2
HPR		VGYVSGWGQSDNFK	772.3620	2	34.3
LCAT	Lecithin:cholesterol acyltransferase	SSGLVSNAPGVQIR	692.8810	2	29.5
LCAT		STELCGLWQGR	653.8140	2	36.0
LCAT		TYIYDHGFPYTDPVGVLYEDGDDTVATR	1060.4875	3	42.1
LPA	Apolipoprotein(a)	GTYSTTVTGR	521.7620	2	20.3

LPA		NPDAVAAPYCYTR	749.3430	2	28.5
LPA		TPENYPNAGLTR	666.8308	2	24.9
PON1	Paraoxonase/arylesterase 1	EVQPVELPNCNLVK	819.9296	2	33.9
PON1		IFFYDSENPPASEVLR	942.4623	2	40.9
PON1		IQNILTEEPK	592.8300	2	30.4
PON3	Paraoxonase/arylesterase 3	ILIGTVFHK	514.3190	2	34.4
PON3		STVEIFK	412.2320	2	31.1
RBP4	Retinol Binding Protein 4	GNDDHWIVDTDYDTYAVQYSCR	898.3790	3	38.3
RBP4		YWGVASFLQK	599.8160	2	42.5
SAA1	Serum Amyloid A1	FFGHGAEDSLADQAANEWGR	726.6590	3	36.7
SAA4	Serum Amyloid A4	AYWDIMISNHQNSNR	924.9259	2	36.5
SAA4		FRPDGLPK	465.2638	2	23.5
SAA4		GPGGVWAAK	421.7296	2	25.2
SERPINA1	Alpha-1-antitrypsin	LSITGTYDLK	555.8060	2	33.4
SERPINA1		SVLGQLGITK	508.3110	2	36.2
TTR	Transthyretin	AADDTWEPFASGK	697.8148	2	35.7
VTN	Vitronectin	SIAQYWLGCPAPGHL	835.4114	2	42.5

Supplemental Table 3. PRM Analysis of HDL Proteins in CKD Patients with or without CVD Events.

Proteins	Description	Control	CVD	P-value	Q-value
**PON3**	**Paraoxonase/arylesterase 3**	**1±0.76**	**0.59±0.55**	**0.0024**	**0.076**
**PON1**	**Paraoxonase/arylesterase 1**	**1±0.56**	**0.71±0.36**	**0.0061**	**0.077**
**APOC3**	**Apolipoprotein C-III**	**1±0.45**	**1.21±0.47**	**0.0097**	**0.077**
**LCAT**	**Lecithin:cholesterol acyltransferase**	**1±0.49**	**0.79±0.35**	**0.010**	**0.077**
**APOA4**	**Apolipoprotein A-IV**	**1±0.46**	**0.80±0.43**	**0.016**	**0.095**
**APOA1**	**Apolipoprotein A-I**	**1±0.28**	**0.86±0.24**	**0.018**	**0.095**
SAA1	Serum Amyloid A1	1±1.28	1.94±3.12	0.040	0.18
APOE	Apolipoprotein E	1±0.43	1.35±0.98	0.089	0.34
VTN	Vitronectin	1±0.70	0.77±0.44	0.14	0.41
APOC1	Apolipoprotein C-I	1±1.56	1.23±1.20	0.14	0.41
SAA4	Serum Amyloid A4	1±0.42	1.08±0.36	0.15	0.41
B2M	Beta-2-Microglobulin	1±0.63	1.39±1.12	0.16	0.41
APOD	Apolipoprotein D	1±1.14	0.82±1.31	0.17	0.41
APOC2	Apolipoprotein C-II	1±0.49	1.15±0.54	0.20	0.44
RBP4	Retinol Binding Protein 4	1±0.41	1.24±0.77	0.21	0.44
APOB	Apolipoprotein B	1±0.78	1.22±1.02	0.23	0.45
HPR	Haptoglobin-related protein	1±0.98	0.97±1.11	0.26	0.45
GC	Vitamin D-binding protein	1±0.89	0.80±0.72	0.27	0.45
APOM	Apolipoprotein M	1±0.66	1.10±0.62	0.27	0.45
CAMP	Cathelicidin Antimicrobial Peptide	1±0.65	1.14±0.67	0.35	0.54
APOC4	Apolipoprotein C-IV	1±1.54	1.24±1.64	0.37	0.54
APOA2	Apolipoprotein A-II	1±0.34	1.03±0.30	0.40	0.56

APOF	Apolipoprotein F	1±0.76	1.14±1.08	0.47	0.64
TTR	Transthyretin	1±0.85	0.95±0.88	0.51	0.65
CLU	Clusterin	1±0.40	1.07±0.52	0.53	0.65
APOL1	Apolipoprotein L-I	1±1.41	0.67±0.72	0.57	0.66
AMBP	Alpha-1-Microglobulin/Bikunin Precursor	1±0.81	1.20±1.08	0.57	0.66
SERPINA1	Alpha-1-antitrypsin	1±0.57	1.01±0.80	0.73	0.81
CFD	Complement Factor D	1±1.06	0.89±0.86	0.88	0.93
CST3	Cystatin C	1±1.05	0.93±0.96	0.90	0.93
LPA	Apolipoprotein(a)	1±1.36	1.31±1.98	0.98	0.98

HDL was isolated from plasma of 46 CKD subjects without CVD events (control) and 46 CKD subjects with CVD events (CVD). Following the digestion of HDL with trypsin, the tryptic digests of HDL proteins were analyzed by isotope dilution targeted MS/MS with PRM. The average levels of proteins in HDL isolated from control group were set as an arbitrary unit of one. Data shown are mean ± SD. Because the levels of proteins in HDL are not normally distributed even after log-transformation, P-values were obtained by a Mann-Whitney non-parametric test. Q-value is the Benjamini-Hochberg adjusted P value. Proteins shown in bold indicate those that are significant when controlling the Benjamini-Hochberg false discovery rate adjusted P-value (Q-value) at 10%.

Supplemental Table 4. Correlations (r) of HDL Proteins and HDL-C with eGFR

Proteins or HDL-C	r	P-value	Proteins	r	P-value
B2M	-0.54	3.8E-08	APOA1	0.046	0.66
AMBP	-0.51	2.6E-07	APOA4	-0.34	7.8E-04
RBP4	-0.42	2.6E-05	APOC3	-0.021	0.85
CFD	-0.36	4.2E-04	LCAT	0.087	0.41
CST3	-0.33	0.0012	PON1	-0.054	0.61
HDL-C	0.11	0.28	PON3	0.0085	0.94

Pearson’s coefficients (r) and p-values are from correlation analysis between the levels of HDL proteins or HDL-C and eGFR.

AMBP, Alpha-1-Microglobulin/Bikunin Precursor; B2M, Beta-2-Microglobulin; CFD, Complement Factor D; CST3, Cystatin C; RBP4, Retinol Binding Protein 4; HDL-C, HDL cholesterol.

Supplemental Table 5. Odds Ratio of HDL proteins and HDL-C for incident CVD

Parameter	OR	95% CI		P-value
difAPOA1	0.48	0.25	0.92	0.026
difAPOA4	0.46	0.24	0.87	0.018
difAPOC3	1.99	1.00	3.96	0.050
difLCAT	0.51	0.26	0.98	0.044
difPON1	0.38	0.17	0.82	0.013
difPON3	0.38	0.18	0.80	0.011
difHDL-C	0.66	0.35	1.23	0.19

Unadjusted odds ratios and P-values are obtained from a one-to-one matched Multinomial Logistic Regression analysis. The difference of levels of an HDL protein or HDL-C between one-to-one matched subjects (e.g. difAPOA1) is used as a variate in the matched logistic regression analysis. Odds ratios are per SD increase of differences of HDL proteins or HDL-C between matched subjects. APOA1, apolipoprotein A-I; APOA4, apolipoprotein A-IV; APOC3, apolipoprotein C-III; LCAT, lecithin:cholesterol acyltransferase; PON1, paraoxonase/arylesterase 1; PON3, paraoxonase/arylesterase 3; HDL-C, HDL cholesterol.

Supplemental Table 6. Odds Ratio of HDL Proteins and HDL-C for Incident CVD after adjusting for clinical confounders.

Parameter	OR	95% CI		P-value
difAPOA1	0.43	0.21	0.91	0.027
difAPOA4	0.41	0.19	0.92	0.031
difAPOC3	2.53	1.06	6.03	0.037
difLCAT	0.51	0.26	1.02	0.055
difPON1	0.38	0.13	0.83	0.019
difPON3	0.36	0.15	0.83	0.017
difHDL-C	0.70	0.36	1.38	0.31

Odds ratios and P-values are obtained from a one-to-one matched Multinomial Logistic Regression analysis after adjusting for clinical confounders, including age, hypertension, present smoker, statin use, BMI, and eGFR. The difference of levels of an HDL metrics between one-to-one matched subjects (e.g. difAPOA1) is used as a covariate in the matched logistic regression analysis. Odds ratios are per SD increase of differences of HDL metrics between matched subjects. APOA1, apolipoprotein A-I; APOA4, apolipoprotein A-IV; APOC3, apolipoprotein C-III; LCAT, lecithin:cholesterol acyltransferase; PON1, paraoxonase/arylesterase 1; PON3, paraoxonase/arylesterase 3; HDL-C, HDL cholesterol.

Supplemental Table 7. Odds Ratio of HDL Proteins for Incident CVD after Adjusting for Clinical Confounders and Lipids Levels.

Parameter	OR	95% CI		P-value
difAPOA1	0.48	0.22	1.05	0.066
difAPOA4	0.42	0.18	0.98	0.045
difAPOC3	2.53	0.91	7.09	0.076
difLCAT	0.47	0.22	0.98	0.043
difPON1	0.38	0.13	0.91	0.031
difPON3	0.36	0.15	0.88	0.024

Odds ratios and P-values are obtained from a one-to-one matched Multinomial Logistic Regression analysis after adjusting for clinical confounders (age, hypertension, present smoker, statin use, BMI, and eGFR) and lipids levels (HDL-C, LDL-C, and triglycerides). The difference of levels of an HDL metrics between one-to-one matched subjects (e.g. difAPOA1) is used as a covariate in the matched logistic regression analysis. Odds ratios are per SD increase of differences of HDL metrics between matched subjects. APOA1, apolipoprotein A-I; APOA4, apolipoprotein A-IV; APOC3, apolipoprotein C-III; LCAT, lecithin:cholesterol acyltransferase; PON1, paraoxonase/arylesterase 1; PON3, paraoxonase/arylesterase 3.

Supplemental Table 8. Odds Ratio of HDL Proteins for Incident CVD after Adjusting for Clinical Confounders, Lipids Levels, proteinuria and CRP.

**Parameter**	**OR**	**95% CI**		**P-value**
difAPOA1	0.44	0.19	1.01	0.053
difAPOA4	0.43	0.18	1.02	0.057
difAPOC3	3.04	0.94	9.83	0.064
difLCAT	0.30	0.11	0.83	0.020
difPON1	0.38	0.03	0.74	0.020
difPON3	0.13	0.03	0.63	0.011

Odds ratios and P-values are obtained from a one-to-one matched Multinomial Logistic Regression analysis after adjusting for clinical confounders (age, hypertension, present smoker, statin use, BMI, and eGFR), lipids levels (HDL-C, LDL-C, and triglycerides), proteinuria, and CRP. The difference of levels of an HDL metrics between one-to-one matched subjects (e.g. difAPOA1) is used as a covariate in the matched logistic regression analysis. Odds ratios are per SD increase of differences of HDL metrics between matched subjects. APOA1, apolipoprotein A-I; APOA4, apolipoprotein A-IV; APOC3, apolipoprotein C-III; LCAT, lecithin:cholesterol acyltransferase; PON1, paraoxonase/arylesterase 1; PON3, paraoxonase/arylesterase 3.
